# Lean integration: A blueprint for occupational health services transformation in healthcare mergers

**DOI:** 10.1177/08404704251360226

**Published:** 2025-09-18

**Authors:** Shaindel Kestenberg, Radhika H. Patel, Adrienna O. Tan, Danielle C. De Graeve, Arash Dhar, Tamara Dus

**Affiliations:** 1 7989 University Health Network, Toronto, Ontario, Canada

## Abstract

This is a case study about University Health Network (UHN) and West Park (WP) Healthcare Centre’s merger in April 2024, marking a significant milestone in organizational transformation. As part of this integration, Occupational Health departments at both organizations were unified into a single team. Data collection, process mapping, and gap analysis were employed to conduct current-state assessments, which identified key differences in organizational structure, database systems, technology platforms, and operational processes. By addressing these gaps, the team clarified roles, centralized infrastructure, aligned policies, and standardized workflows. Four key domains were targeted for integration: organizational structure, database systems, technology platforms, and operational processes. Challenges in change management, resource allocation, and training were addressed strategically. This integration approach improved multidisciplinary communications, standardized protocols, reduced manually intensive administrative workload, and enhanced safety, emphasizing project scoping, cross-functional collaboration, and innovative solutions for operational excellence.

## Introduction

The purpose of the case study is to support Canadian healthcare organizations with a scalable model for mergers and change management using Lean methodologies. In April 2024, University Health Network (UHN), a multi-site healthcare and medical research organization employing over 27,000 members, voluntarily merged with West Park Healthcare Centre (WP), a specialized organization providing rehabilitative and complex care services with approximately 1,300 healthcare workers and volunteers.

Occupational Health is essential for employee well-being, workplace safety, and organizational efficiency. A strong framework is required to uphold regulatory and legal obligations that will create a safe, cohesive, and productive workplace environment. UHN and WP completed a strategic initiative to consolidate and align Occupational Health Services (OHS) operations across both organizations.

Prior to the merger, UHN and WP were different hospital systems, each maintaining separate organizational frameworks; database systems; technologies; and approaches to managing clinics, disability management, wellness, and safety services. These differences led to challenges in communication, data uniformity, and cross-site collaboration, showing the need for a carefully planned integration strategy.

Scholarly resources highlight different frameworks and strategies to support effective change management during transitions.^1-3^ The effectiveness of each approach depends on the organizational culture and operational landscape of the merging organizations.^4^

The OHS integration strategy was grounded in core Lean principles:

(1) Specify value – defining high-value tasks and essential workflows.^5^
(2) Value stream mapping – operational workflows to flag and eliminate non-value-added waste (Muda) through automation and digitization.^5^
(3) Flow – streamlining and standardizing processes across sites.^5^
(4) Pull – allocating resources based on real-time needs for efficient operations.^5^
(5) Continuous improvement (Kaizen) – embedding feedback loops to support ongoing changes, facilitate staff engagement, and enable real-time adjustments.^5^

The effectiveness of Lean methodologies to refine processes and reduce wasteful steps has improved operational efficiency and quality of care across hospital settings.^6-9^ Applying these principles, the project team was able to document existing pain points, develop targeted interventions, and lay groundwork for long-term improvement.

## Multi-method approach

A multi-method approach was used to guide the integration of OHS across both organizations. The planning for OHS integration process began in June 2024, and the integration was completed by January 2025. This required no additional Human Health Resources (HHR) to complete but required dedicated and focused time to complete the three key phases below.

(1) Data collection: Information was gathered through multiple sources including system audits, contracts, policies, document reviews, interviews, and unit observations (Gemba walks) at WP to observe daily activities and collect feedback from key stakeholders.^9^
(2) Process mapping: Value stream mapping exercises were completed for onboarding, incident reporting, disability management, clinical operations, and other core OHS functions during Gemba walks to create detailed workflow maps.
(3) Gap analysis: A comparative analysis of systems, applications, and procedures was conducted to identify similarities and differences in role structures, database systems, technology platforms, and operational models.

## Findings

The multi-method approach established a foundational understanding of operational inefficiencies, redundancies, and opportunities for harmonization. Four key areas required strategic alignment: organizational structure, database systems, technology platforms, and operational workflows.

### Organizational structure integration

**Figure 1.**
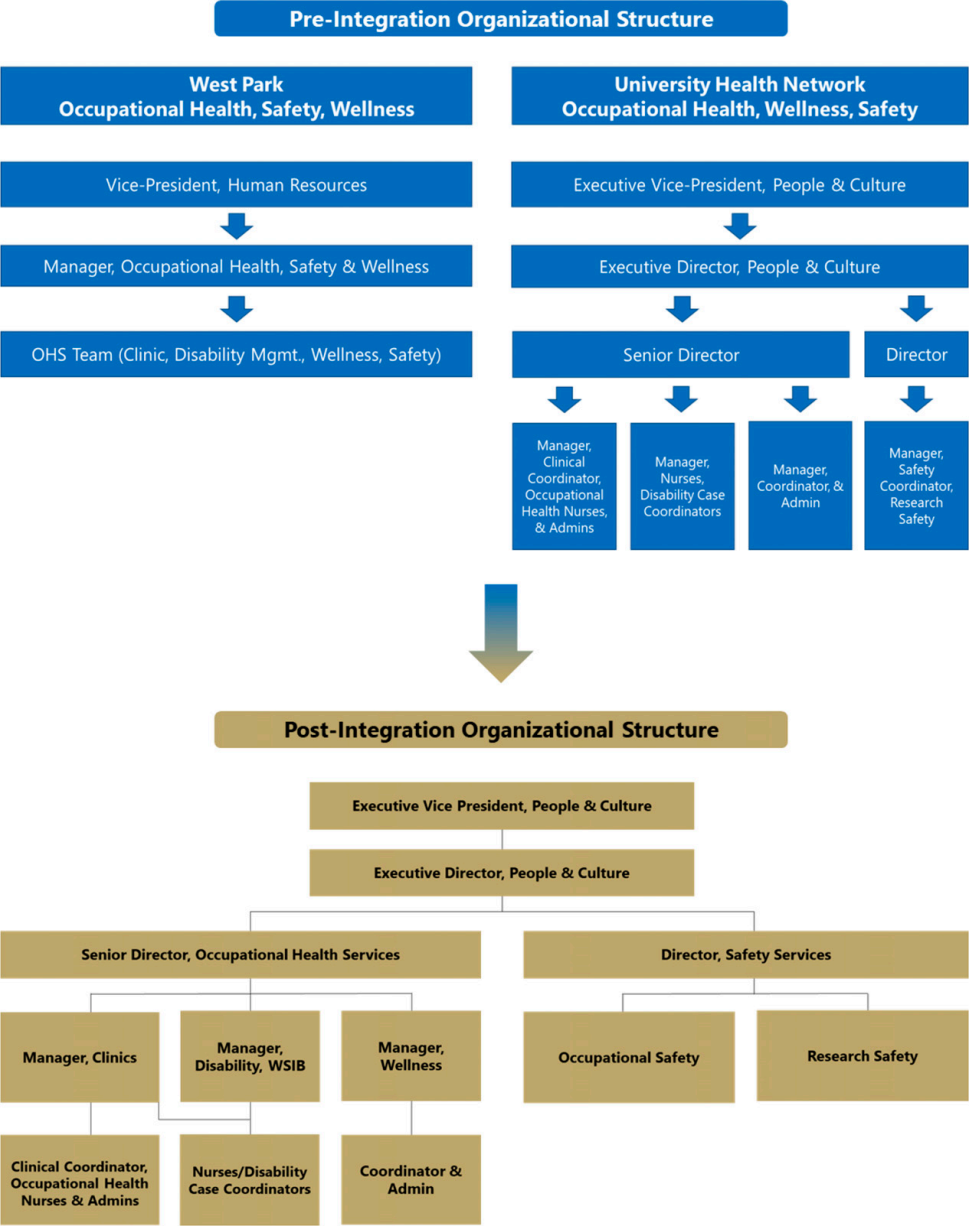
Conceptual comparison of pre-integration and post-integration organizational structures.

Before integration, WP’s Occupational Health, Safety, and Wellness were centralized, while UHN operated under a segmented model with specialized teams dedicated to each core area of OHS. Following the integration, WP adopted UHN’s team-based model, aligning staff into specialized teams based on their roles (Figure 1). This restructuring enhanced role clarity, distinguished team workflows, and fostered supportive collaboration across Clinics, Disability Management, Wellness, and Safety Services teams.

In this new model, the Clinics team oversees clinic appointments, onboarding, and first aid for occupational injuries; the Disability Management team handles short-term disability claims and coordinates return-to-work processes; the Wellness team promotes employee health and manages workplace wellness programs; and the Safety Services team is responsible for incident reporting and ensuring adherence with safety protocols and Ministry of Labour requirements.

This model shifts towards a streamlined, specialized service delivery across the newly integrated organization.

### Employee health record database integration

Following the current-state assessment of system features and applications used within the UHN OHS team, multiple solutions were considered to determine the best route for database integration between UHN and WP (Table 1). Solutions were evaluated based on operational efficiency, technical security, feasibility, and implementation timeline. A fully integrated database system with unified modules and webform applications emerged as the most valuable long-term solution. Despite a longer implementation period, this solution standardizes and unifies all OHS operations across one shared platform, ultimately offering greater sustainability and scalability to future organizational growth and system changes.

**Table 1.** Proposed solutions summary for employee health record database system alignment between UHN and WP.

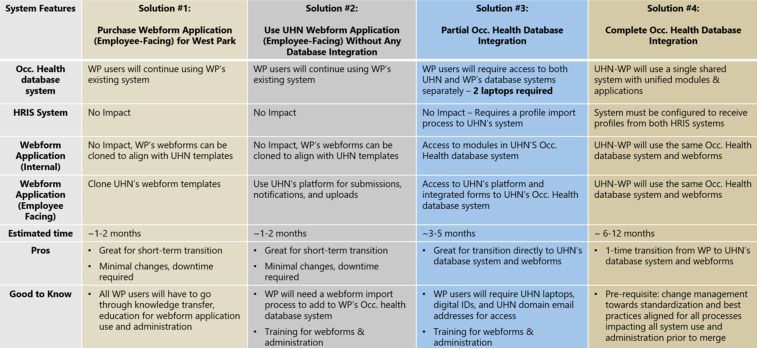

### Technology integration

Comparative analysis was done to examine disparities in technology platforms used across both organizations. WP relied on independent third-party platforms and applications for different operational workflows including staff communications, reporting, file storage, and data management, preventing seamless data flow and system synchronization. In contrast, UHN utilized different Microsoft 365 suite applications to centralize all functions into one single platform. The integration of Microsoft 365 integration was identified as the key solution to improve communication channels, secure information sharing and collaboration, and streamline access to department resources, ultimately removing inconsistencies and supporting timely decision-making.

Further assessment of the employee reporting infrastructure across both organizations revealed that UHN utilizes a centralized, employee-facing webform application for onboarding, communicable symptom reporting, immunization submissions, absence reporting, incident reporting, and return-to-work declaration whereas WP relied on a mix of PDF forms, manual data entry, and external portals, resulting in fragmented workflows. Leveraging UHN’s existing streamlined reporting infrastructure to align with and integrate WP’s processes was identified as the solution.

### Alignment of operational workflows

A critical component of this merger involved departmental review and operational assessment to align key workflows, reduce variation, and standardize practices. Through process mapping events held with both teams, three key clinic operations were identified to assess pain points and create actionable plans to align operations.

**Figure 2.**
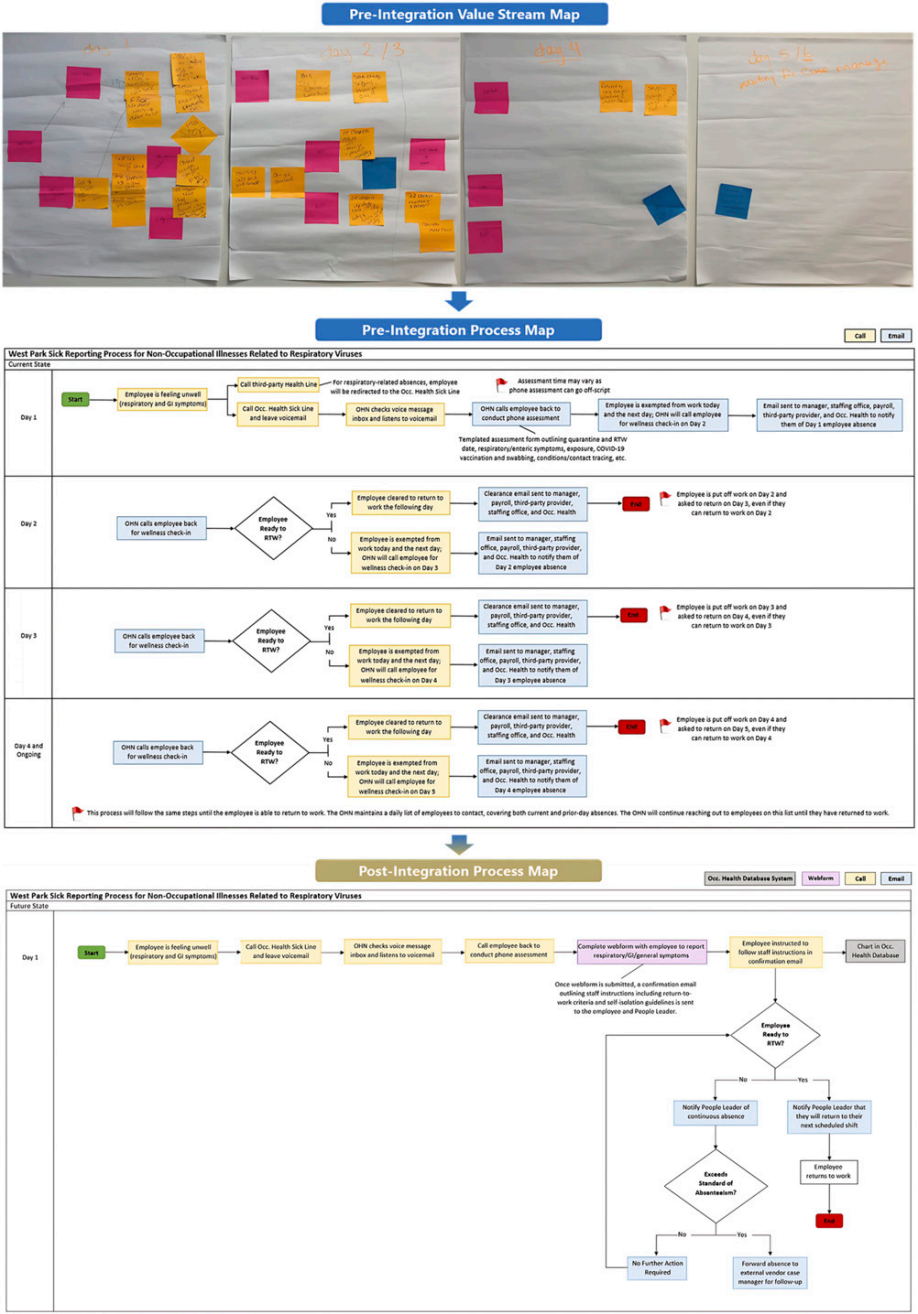
Pre-integration value stream map and process map, followed by the post-integration process map for the sick line reporting process at WP.

(a) Sick reporting was the first process to be integrated. In December 2024, the WP OHS follow-up process for communicable symptoms was realigned to mirror UHN OHS procedures. Under this updated approach, OHS no longer manages routine respiratory or gastrointestinal absences. Instead, employees report symptoms of a potentially communicable absence to the OHS sick phone line on the first day of absence. This notification is solely for the purpose of monitoring trends and identifying potential workplace transmission. Staff are expected to notify the designated third-party provider for sick case management. If an absence exceeds 3 days, the third-party case manager will initiate disability case management. Furthermore, OHS nurses no longer need to conduct follow-up, or wellness calls to determine Return-To-Work (RTW) eligibility. Employees may return to work once their respiratory symptoms have improved, they meet the symptom resolution criteria, and they have completed an electronic attestation confirming their readiness to return. To support this process, automated email notifications, powered by digital algorithms guide both employees and People Leaders through each step, streamlining communication, reinforcing accountability, and empowering staff with clear responsibilities (Figure 2).
(b) New employee onboarding process was the second workflow that was redesigned to enhance efficiency and restructure staffing responsibilities. Administrative staff now oversee compliance follow-ups under nursing supervision, enabling nurses to focus on clinical care to better align to their professional scope. Immunization records are submitted through a centralized webform rather than email, simplifying data collection, administrative burden, and reducing delays. People Leaders receive automated updates on staff compliance, supported by a formal review and clearance process. Clinic visits are now appointment-based and booked online to improve scheduling. Escalations for non-compliance are directed through People Leaders instead of People & Culture. Together, these changes support a more coordinated, automated, and accountable onboarding experience.
(c) The final task was to align the clinic setting and functions, removing involvement in processes unrelated to OHS. Clinic operations have been refocused to prioritize occupational health in the workplace. The clinic now concentrates on organizational priorities such as workplace incident follow-ups, exposure assessments, outbreak management, and onboarding immunizations. Payroll and labour relations issues were redirected to their respective departments. Over-the-counter medications are no longer dispensed by OHS and are provided directly through pharmacy. These changes redirected task ownership to appropriate departments, enabling OHS staff to clarify roles and focus on duties aligned within UHN’s OHS service scope.

## Implementation strategy

To support the integration of OHS at UHN and WP, a structured training program ran from October 2024 to January 2025, ensuring WP OHS staff were equipped to adjust current practices and adopt new workflows. The training curriculum blended in-person shadowing, self-directed module learning, and hands-on clinical practice to review clinics and disability management processes including onboarding, communicable disease management, safety incident reporting, cold chain maintenance, and Workplace Safety Insurance Board (WSIB) procedures. Nurses also participated in shadowing and on-site instruction at downtown UHN locations to gain real-time exposure to standardized practices. By March 2025, WP nurses joined the UHN on-call rotation—promoting consistency and collaboration across sites. This strategy supported a smooth operational transition and reinforced change management practices across clinical teams.

The training program and broader integration strategy were intentionally aligned with established change management principles. National Change Management Framework identifies six essential elements: governance and leadership; stakeholder engagement; communications; training and education; workflow analysis and integration; and monitoring and evaluation as key to implementing and sustaining organizational change.^10^ These principles were embedded throughout the transition through structured leadership involvement, early frontline engagement, consistent communication strategies, and continuous feedback mechanisms.

## Discussion

### Streamlined operations

Through Gemba walks and value stream mapping, UHN and WP’s OHS teams gained a strong understanding of current-state operations, identifying key similarities, gaps, and opportunities for improvement. These insights informed a targeted strategy to streamline OHS across multiple domains. The organizational structure was refined by adopting OHS’s team-based model, enhancing role clarity and accountability. A unified database system with standardized webform applications replaced fragmented data sources, enabling consistent documentation and efficient navigation. Technological integration consolidated core functions including communication, appointment scheduling, file storage, and document sharing into a centralized Microsoft 365 platform to improve cross-site coordination. Core workflows such as new employee onboarding were aligned to UHN’s standardized procedures, introducing automation, clear escalation pathways, and real-time compliance tracking. These outcomes align with Lean principles of flow and value stream mapping. The analysis of organizational context, operational characteristics, and technical factors in diverse healthcare settings can influence the harmonization of systems, improving resource utilization and workflow standardization across healthcare teams.^11-13^ Streamlining efforts have replaced disconnected processes with a cohesive and efficient OHS delivery model.

### Clarity on role and responsibilities

The integration established clear role delineation across teams and departments by restructuring responsibilities and eliminating waste. For example, onboarding tasks were clearly defined within the team, where administrative staff managed compliance tracking, while nurses focused on clinical assessments. Absence reporting was streamlined to eliminate repetitive nursing follow-ups, reduce communication gaps between appropriate parties, and decrease the amount of time for return-to-work clearance. These efforts provided role clarity and allowed respective staff to focus on high-value tasks that fall within scopes of practice. Moreover, clinic operations were redefined to focus on occupational incidents, exposures, and outbreaks, while non-occupational tasks were identified and redirected to appropriate departments. These changes have reduced turnaround times, improved training and education, streamlined assessments, and standardized care, ensuring meaningful contributions across OHS functions. The efficacy of resource allocation and waste management is critical in improving care delivery across patient care and research settings.^6-8,11-15^ These outcomes reflect key Lean principles of value and pull where high-impact work is aligned with roles and initiated based on demand and eliminated process waste.

### Improved change management and communication

This case study emphasized the importance of organizational structure alignment and communication. Effective change management in healthcare requires structural redesign and in-depth reviews. This includes initial data collection, gap analysis, and post implementation results showing improvement. Engagement from multiple teams, psychological readiness, and leadership support is critical for success. Regular updates outlining key milestones were shared with staff, and leadership forums were conducted to encourage two-way communication and feedback to continuously improved processes with real-time adjustments. Inclusive communication, leadership commitment, and frontline engagement play important roles in change management.^11,16^ It is evident that successful healthcare mergers require structured evaluation frameworks and ongoing assessment to preserve care quality, highlighting the implicit need for coordinated leadership, transparent communication, and strategic organizational support throughout the integration process.^17^ Providing targeted resources and training ensured that frontline staff were engaged during changes and transitions, ultimately reducing resistance and fostering long-term adoption.

### Cost-saving measures and health human resource considerations

The strategic consolidation of roles and operations has enhanced budgeting and Health Human Resource planning. Staff were realigned based on scope and function, with nurses focusing on clinical care, disability case coordinators supporting return-to-work programs, and safety services on leading the development of safe work practices and standards. This realignment reduced role overlap and allowed staff to work at their full scope. The enhancements and efficiencies eliminated the necessity to renew two temporary contract positions. Operationally, the discontinuation of non-occupational services created space for higher value work. The implementation of process automation and digitization already in effect at UHN, improved the service delivery model. These changes demonstrate Lean waste reduction across human resource, financial, and operational domains.

Overall, the integration embodied the Lean principle of Kaizen, emphasizing continuous improvement to ensure a smooth transition and sustainable alignment.^18^ By streamlining processes, clarifying roles, adopting cost-saving measures, and implementing change management practices, the integrated OHS model is well-positioned to support future growth and innovation opportunities.

### Limitations

This case study has three limitations. First, the integration was conducted within a small department of fewer than 60 employees, which may limit applicability in larger settings. Second, the successful implementation relied on the team’s existing knowledge of Lean change management principles, which may not be present in other teams. Third, active engagement from leadership and frontline staff was essential to the integration’s success and replicating this with limited engagement may yield different outcomes.

## Conclusion

Given the limited literature available currently to support healthcare organizations with mergers, we believe that UHN-WP’s migration case study and lessons learned serve as a blueprint for future mergers and migrations and provides a scalable model.

The Lean project methodology focused on identifying organizational disparities, process and resource waste, and workflow assessments to develop a unified service delivery model.

Occupational Health plays an important role in a healthcare organization, directly influencing employee well-being, employee safety, and overall organizational effectiveness. A strong framework is essential for meeting regulatory requirements and laws, and for cultivating a safe, cohesive, and efficient workplace culture. Lean practices have reinforced a sustainable OHS framework across the merged organization, positioning it for long-term scalability and impact. In this context, UHN and WP Healthcare Centre successfully completed an organizational merger.

## References

[1] Klar B . Health system integration: prescription for success. Healthc Financ Manag. 2018. https://www.hfma.org/finance-and-business-strategy/partnerships-and-value/60449/. Accessed June 5, 2025.

[2] Maile EJ Mitra M Ovseiko P Dopson S . Merger and post-merger integration at Oxford University Hospitals: mixed-methods evaluation and lessons learned. J Health Organ Manag. 2022;36(4):503-520. doi:10.1108/JHOM-01-2021-0024 35015386

[3] Moeller S Faelten A Ajayi D . Mergers in the NHS: Lessons Learnt and Recommendations. Cass Business School; 2016. https://ddavisconsulting.com/wp-content/uploads/2016/11/Mergers_Cass_full_report.pdf. Accessed June 5, 2025.

[4] Hall K . Successful merger integration: a strategic approach. Kaufman Hall. 2020. https://www.kaufmanhall.com/insights/article/successful-merger-integration-strategic-approach. Accessed June 5, 2025.

[5] Womack JP Jones DT . Lean thinking—banish waste and create wealth in your corporation. J Oper Res Soc. 1997;48(11):1148. doi:10.1057/palgrave.jors.2600967

[6] Ng D Vail G Thomas S Schmidt N . Applying the Lean principles of the Toyota Production System to reduce wait times in the emergency department. CJEM. 2010;12(1):50-57. doi:10.1017/s1481803500012021 20078919

[7] Lan M Lam J Robinson S Pozzobon L . 37 The road to minimizing waste in the review of potential critical patient incidents by patient safety specialists. BMJ Open Qual. 2025;14(Suppl 3):A25. doi:10.1136/bmjoq-2025-QSHU.37

[8] Sloan T Fitzgerald A Hayes KJ Radnor Z Robinson S Sohal A . Lean in healthcare – history and recent developments. J Health Organ Manag. 2014;28(2):130-134. doi:10.1108/JHOM-04-2014-0064 25065106

[9] Aij KH Teunissen M . Lean leadership attributes: a systematic review of the literature. J Health Organ Manag. 2017;31(7-8):713-729. doi:10.1108/JHOM-12-2016-0245 29187082 PMC5868554

[10] National Change Management Framework . Canada Health Infoway. Infoway-inforoute.ca. Published 2025. https://www.infoway-inforoute.ca/en/component/edocman/change-management/565-national-change-management-framework?Itemid=101. Accessed June 26, 2025.

[11] Veres C Stoian M Szabo DA Gabor MR . The role of leadership in lean healthcare transformation: a mixed-methods study. J Knowl Econ. 2025; Published online March 19. doi:10.1007/s13132-025-02661-5

[12] Hung DY Harrison MI Martinez MC Luft HS . Scaling Lean in primary care: impacts on system performance. Am J Manag Care. 2017;23(3):161-168. 28385026

[13] Mishra V Sharma MG . Factors affecting implementation of digital lean in healthcare in the post-COVID world – mixed-method approach. TQM J. 2024;36(6):1651-1664. doi:10.1108/TQM-08-2023-0251

[14] Tlapa D Zepeda-Lugo CA Tortorella GL , et al. Effects of lean healthcare on patient flow: a systematic review. Value Health. 2020;23(2):260-273. doi:10.1016/j.jval.2019.11.002 32113632

[15] De OKB dos Santos EF Garcia Junior LV . Lean healthcare as a tool for improvement: a case study in a clinical laboratory. In: Duffy VG Lightner N , eds. Advances in Human Factors and Ergonomics in Healthcare, Vol. 482. Springer; 2017:129-140. doi:10.1007/978-3-319-41652-6_13

[16] Best A Greenhalgh T Lewis S Saul JE Carroll S Bitz J . Large-system transformation in health care: a realist review. Milbank Q. 2012;90(3):421-456. doi:10.1111/j.1468-0009.2012.00670.x 22985277 PMC3479379

[17] Mariani M Sisti LG Isonne C , et al. Impact of hospital mergers: a systematic review focusing on healthcare quality measures. Eur J Publ Health. 2022;32(2):191-199. doi:10.1093/eurpub/ckac002 PMC9090279 35157040

[18] Morell-Santandreu O Santandreu-Mascarell C García-Sabater J . Sustainability and Kaizen: business model trends in healthcare. Sustainability. 2020;12(24):10622. doi:10.3390/su122410622

